# Single-Session Versus Hypofractionated Gamma Knife Radiosurgery for Large Parasagittal Meningiomas: A Retrospective Comparative Study

**DOI:** 10.3390/cancers18193188

**Published:** 2026-10-02

**Authors:** Mehmet Ali Tepebasili, Amir Salar Nazari, Ali Haluk Duzkalir, Dogu Cihan Yildirim, Mehmet Orbay Askeroglu, Mert Veznikli, Selcuk Peker

**Affiliations:** 1 Department of Neurosurgery, School of Medicine, Koc University, 34450 Istanbul, Turkey; mtepebasili@ku.edu.tr; 2 School of Medicine, Medipol University, 34810 Istanbul, Turkey; amir.nazari@std.medipol.edu.tr; 3 Gamma Knife Center, Koc University Hospital, 34025 Istanbul, Turkey; aduzkalir@kuh.ku.edu.tr (A.H.D.); doyildirim@kuh.ku.edu.tr (D.C.Y.); maskeroglu@kuh.ku.edu.tr (M.O.A.); 4 Department of Biostatistics, School of Medicine, Koc University, 34450 Istanbul, Turkey; mveznikli@ku.edu.tr

**Keywords:** parasagittal meningioma, Gamma Knife radiosurgery, hypofractionated radiosurgery, stereotactic radiosurgery, local control, peritumoral edema, superior sagittal sinus, large meningioma

## Abstract

**Background/Objectives:** Large parasagittal meningiomas (PSMs) present significant surgical challenges due to their proximity to the superior sagittal sinus, and the optimal radiosurgical strategy for large tumors remains debated. This study compared local control (LC) and treatment-related radiological and clinical outcomes after single-session (ss-GKRS) and hypofractionated (hf-GKRS) Gamma Knife radiosurgery in PSMs ≥8 cm^3^. **Methods:** Eighty-seven patients with 87 radiologically confirmed PSMs treated between April 2006 and February 2025 were reviewed. Patients with histopathologically confirmed atypical or anaplastic meningiomas or imaging features suspicious for higher-grade disease were excluded. Fifty-nine patients underwent ss-GKRS and 28 received hf-GKRS. The median tumor volume was 11.2 cm^3^ (range 8–37.8 cm^3^). LC was defined as tumor volume regression or stability on serial MRI. Radiological complications were defined as new or worsening peritumoral T2 hyperintensity, and clinical complications as new neurological symptoms. Time to local progression was compared using the log-rank test, with an exploratory volume cut point from ROC analysis. **Results:** The median follow-up was 62 months (range 12–186 months). Overall crude LC was 93.1%, including 89.8% (53/59) after ss-GKRS and 100% (28/28; exact 95% CI 87.7–100%) after hf-GKRS. All six local progressions occurred in the ss-GKRS group; however, freedom from local progression did not differ significantly between the groups (log-rank *p* = 0.137). In an exploratory volume-stratified analysis, LC was 100% in both groups for tumors ≤11.75 cm^3^. Among tumors >11.75 cm^3^, crude LC was 71.4% (15/21) after ss-GKRS and 100% (16/16) after hf-GKRS (Fisher exact *p* = 0.027). Post-treatment radiological edema occurred in 39.0% after ss-GKRS versus 25.0% after hf-GKRS (*p* = 0.235). **Conclusions:** Overall freedom from local progression did not differ significantly between ss-GKRS and hf-GKRS. No local failures were observed after hf-GKRS during its shorter available follow-up, including among tumors >11.75 cm^3^ in an exploratory analysis. These findings support hf-GKRS as a feasible treatment option for selected larger PSMs, but neither treatment superiority nor a definitive tumor-volume threshold can be established from this retrospective cohort.

## Simple Summary

Parasagittal meningiomas (PSMs) are difficult to resect completely because they often involve the superior sagittal sinus, one of the brain’s major veins. As a result, Gamma Knife radiosurgery is commonly used as an alternative or complementary treatment. For larger tumors, single-session treatment may be limited by the risk of radiation-related edema. Dividing the treatment into several sessions may reduce this risk, but there is limited evidence for this approach in parasagittal meningiomas. We reviewed 87 patients with 87 large PSMs (≥8 cm^3^) treated at a single institution and compared outcomes between single-session and multi-session Gamma Knife radiosurgery. Freedom from local progression did not differ significantly between the groups (*p* = 0.137), nor did post-treatment radiological edema (*p* = 0.235) or new clinical symptoms (*p* = 0.744). These findings offer practical guidance for treating large parasagittal meningiomas and highlight the need for prospective studies to determine which patients benefit most from fractionated treatment.

## 1. Introduction

Meningiomas are the most common primary intracranial neoplasm [1]. Parasagittal meningiomas (PSMs), which arise in relation to the superior sagittal sinus (SSS), constitute one of the largest anatomical subgroups, accounting for approximately one fifth to one third of intracranial meningiomas in surgical series [2,3]. Their location presents significant surgical challenges, particularly in cases with invasion of the sagittal sinus or adjacent venous structures, where complete resection may be difficult and the risk of postoperative morbidity is increased [4,5]. The risk of tumor recurrence is markedly higher following subtotal resection (STR), and repeated surgeries further elevate morbidity and mortality. Following STR, adjuvant radiotherapy or radiosurgery may be considered to improve long-term tumor control, particularly when residual or recurrent disease is present [6].

Radiosurgery has been reported to provide effective tumor control rates with minimal complications in meningiomas located in eloquent areas [7,8,9,10]. Although radiosurgery has traditionally been performed as adjuvant therapy after surgical resection or as primary treatment for small- to medium-sized tumors (<3 cm), recent evidence indicates that it can also be a safe and effective therapeutic option for large meningiomas [11,12,13,14].

Radiation-related toxicity after single-session SRS is a recognized concern in larger supratentorial meningiomas [15,16,17]. Hypofractionation may improve normal-tissue tolerance while maintaining tumor control [7], but comparative evidence specific to large PSMs remains limited. We therefore compared freedom from local progression and treatment-related radiological and clinical outcomes after ss- and hf-GKRS in patients with large PSMs.

## 2. Materials and Methods

### 2.1. Ethics Statement

This retrospective study, utilizing prospectively maintained data, was approved by the Institutional Review Board of Koç University and was performed in accordance with the principles of the Declaration of Helsinki. Informed consent for treatment and data collection was obtained from all participants.

### 2.2. Eligibility and Demographic Data

A total of 741 PSM patients who underwent ss-GKRS or hf-GKRS for PSM between April 2006 and February 2025 were screened for eligibility. Of these, 87 patients met the study criteria and were included in the final cohort. The study inclusion criteria were age ≥18 years, radiologically confirmed PSM, treatment with ss-GKRS or hf-GKRS, and adequate follow-up (≥12 months). Patients who developed local progression, new or worsening post-treatment radiological edema, or new neurological symptoms within the first 12 months remained eligible, provided that at least 12 months of clinical and radiological follow-up was available. Patients with histopathologically confirmed atypical or anaplastic meningiomas were excluded from the study. Thirty-six of the included patients had undergone prior surgical resection, and all had histopathologically confirmed WHO grade I meningioma. For patients managed without surgery, a diagnosis of radiologically presumed benign meningioma was established by consensus review of a multidisciplinary team comprising neurosurgery, radiology, and radiation oncology. Only well-circumscribed, extra-axial, dural-based lesions with avid, predominantly homogeneous contrast enhancement and no imaging features suggestive of higher-grade pathology were included. A dural tail and/or CSF cleft were considered supportive but not mandatory features. Patients with atypical imaging characteristics or an uncertain diagnosis were excluded from the study.

To focus on relatively large tumors while preserving an overlapping volume range between treatment groups, a pragmatic minimum tumor volume threshold of ≥8 cm^3^ was selected. In the ss-GKRS group, tumor volumes ranged from 8 to 32 cm^3^, whereas in the hf-GKRS group they ranged from 8 to 37.8 cm^3^. The degree of sinus invasion was graded in accordance with the Sindou classification [18]. Tumor localization was categorized as anterior third, middle third, or posterior third based on predefined anatomical criteria [19]. All data were retrieved from patient charts and neuroimaging databases.

### 2.3. Radiosurgical Technique

ss-GKRS and hf-GKRS were performed with a Leksell Gamma Knife (Elekta Instrument AB, Stockholm, Sweden). ss-GKRS was performed using a Leksell stereotactic frame (Elekta Instrument AB, Stockholm, Sweden), whereas hf-GKRS was performed with a thermoplastic mask. The methodology for frameless hypofractionation is presented in detail in another manuscript [20]. A reference cone beam CT scan was obtained at each session in hypofractionated treatment. Pre-treatment imaging, including 1 mm thin-slice volumetric axial CT, T1-weighted MRI, contrast-enhanced T1-weighted MRI, and T2-weighted MRI, was performed and imported into Leksell GammaPlan version 11.4.2 for both treatment methods. The gross tumor volume was equal to the clinical target volume and the planning target volume. Shots with collimator sizes of 4, 8, and 16 mm were placed inside the target and manipulated to simultaneously achieve high target coverage, high selectivity, a gradient index <3, and reasonable treatment times using the best-fit isodose method, until a clinically acceptable plan was formulated by the radiosurgery team.

For hf-GKRS, patient selection was based on tumor volume, proximity to eloquent cortex, degree of SSS involvement, presence of pre-treatment edema, and estimated risk of radiation-induced adverse effects. All hf-GKRS cases were treated with 3, 5, or 10 fractions. The median prescribed margin dose was 12 Gy (range, 10–15 Gy) in the ss-GKRS group and 20 Gy delivered in fractions (range, 18–30 Gy total) in the hf-GKRS group. The biologically effective dose (BED) was calculated using the linear–quadratic model, with an α/β ratio of 3 Gy for meningioma. Values are reported as BED_3_ [21].

### 2.4. Follow-Up and Outcome Parameters

All patients underwent follow-up with MRI and clinical evaluation six months after treatment, followed by annual assessments thereafter, with intervals adjusted based on clinical status and imaging findings. Follow-up data included LC, adverse radiological findings, new clinical symptoms, and the need for salvage intervention. Tumor response was assessed based on volumetric changes on MRI [22]. Progression was defined as an increase of ≥20% in tumor volume, and regression as a decrease of ≥20%; volume changes of <20% in either direction were classified as stable disease. Tumors showing regression or stable disease were classified as locally controlled, whereas local failure (LF) was defined as progression.

Post-treatment radiological edema was defined as newly developed peritumoral T2 hyperintensity in patients without baseline edema, or unequivocal enlargement of pre-existing peritumoral T2 hyperintensity in patients with baseline edema, in the absence of concurrent tumor progression. For descriptive analyses, this endpoint was also stratified by baseline edema status, distinguishing new edema in patients without pre-treatment edema from worsening of pre-existing edema in those with baseline edema. This definition included both symptomatic cases and incidental findings detected on routine follow-up imaging in the absence of clinical symptoms. Because pre-treatment edema was substantially more frequent in the hf-GKRS group (60.7% versus 15.3%), the comparability of post-treatment edema rates between groups is limited; this is addressed in the Limitations. Clinical complications were defined as new neurological symptoms developing after treatment.

### 2.5. Statistical Analysis

Continuous variables were summarized as medians with ranges and compared between groups using the Mann–Whitney U test, whereas categorical variables were reported as frequencies and percentages. The primary effectiveness endpoint was time to local progression. Freedom from local progression was estimated using the Kaplan–Meier method, with treatment groups compared by the log-rank test; deaths unrelated to PSM were censored at the date of death. LC status (progression versus no progression) was used as a binary endpoint for exploratory subgroup analyses only.

Proportions are reported with exact (Clopper–Pearson) 95% confidence intervals, and categorical comparisons were made using Fisher’s exact test. Because no LFs occurred in the hf-GKRS group, effect-size estimates for the volume-stratified comparisons were considered unstable and are therefore not reported.

Receiver operating characteristic (ROC) curve analysis was performed to assess the discriminative ability of tumor volume for LF, which was specified as the positive state. The cut point maximizing the Youden index (sensitivity + specificity − 1) was identified. The 95% confidence interval for the area under the curve was derived using the method of Hanley and McNeil. This cut point was derived and evaluated within the same dataset without independent or bootstrap validation and is therefore exploratory. The resulting cut point was used only for hypothesis-generating descriptive subgroup analyses and was not considered a validated treatment-selection threshold.

All statistical analyses were performed using IBM SPSS Statistics (v.28.0; IBM Corp., Armonk, NY, USA). A two-sided *p*-value < 0.05 was considered statistically significant, with the explicit caveat that the volume-stratified analyses are exploratory and were not corrected for multiplicity.

## 3. Results

### 3.1. Patient Characteristics

A total of 87 patients with 87 treated PSMs were included (60.9% female). Of these, 59 patients (67.8%) were treated with ss-GKRS and 28 patients (32.2%) with hf-GKRS. ss-GKRS was performed throughout the study period (April 2006–February 2025), whereas hf-GKRS was performed between January 2018 and October 2024. The most common presenting symptom was headache, observed in 40 patients (46.0%), followed by seizures in 29 patients (33.3%). Motor or sensory deficits were present in 9 patients (10.3%), while gait disturbance was noted in 3 patients (3.4%). Four patients (4.6%) were diagnosed incidentally. Aphasia and visual disturbance were each observed in 1 patient (1.1%). The most frequent type of SSS invasion was Sindou type VI (36.8%). Of the tumors, 64 (73.6%) were located in the middle third, 18 (20.7%) in the anterior third, and 5 (5.7%) in the posterior third. Thirty-six patients (41.4%) had a history of prior surgical resection. Pre-treatment edema was present in 26 patients (29.9%) and was markedly more frequent in the hf-GKRS group (60.7%, 17/28) than in the ss-GKRS group (15.3%, 9/59). Median follow-up for the entire cohort was 62 months (range 12–186 months); median follow-up was 71.5 months (range 12–186) after ss-GKRS and 60 months (range 12–98) after hf-GKRS. Eight patients died during follow-up from causes unrelated to PSM and were censored at the date of death. Detailed patient characteristics are summarized in Table 1.

**Figure 1:**
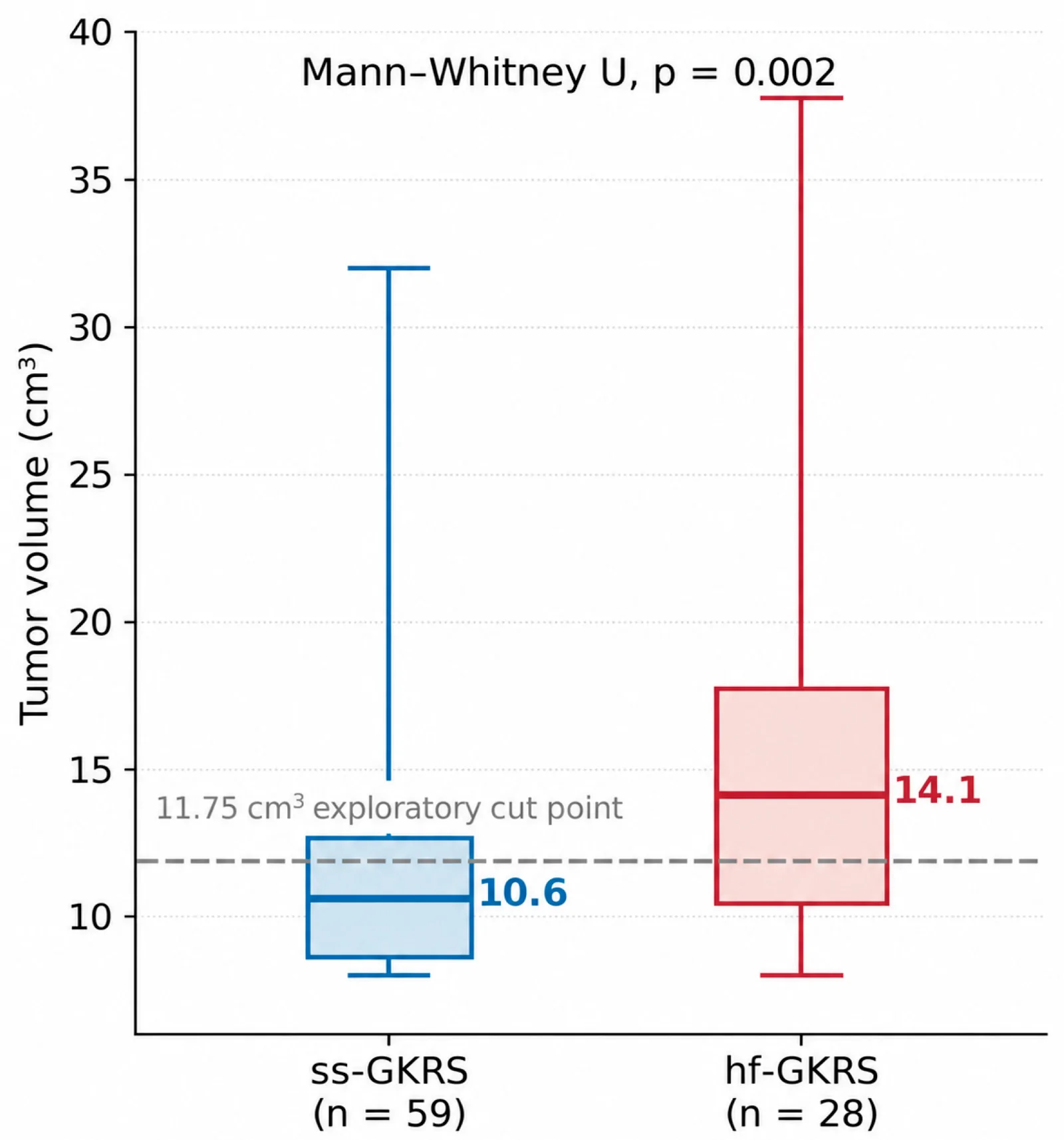
Tumor volume distribution by treatment modality. Boxes show the interquartile range with the median indicated by the horizontal line; whiskers extend to the minimum and maximum observed volumes. Median tumor volume was 10.6 cm^3^ (range 8–32) in the ss-GKRS group and 14.1 cm^3^ (range 8–37.8) in the hf-GKRS group (Mann–Whitney U, *p* = 0.002). The dashed line marks the 11.75 cm^3^ cut point derived by ROC analysis (see Section 2.5). ss-GKRS, single-session Gamma Knife radiosurgery; hf-GKRS, hypofractionated Gamma Knife radiosurgery.

Median tumor volume for the entire cohort was 11.2 cm^3^ (range 8–37.8 cm^3^); it was 10.6 cm^3^ (range 8–32 cm^3^) in the ss-GKRS group and 14.1 cm^3^ (range 8–37.8 cm^3^) in the hf-GKRS group (Mann–Whitney U, *p* = 0.002; Figure 1). Prior surgical resection was also more frequent in the hf-GKRS group than in the ss-GKRS group (64.3% vs. 30.5%, Fisher’s exact *p* = 0.005). Thus, the treatment groups were not balanced with respect to tumor volume or prior surgery. Median BED_3_ was 60.0 Gy_3_ (range 43.3–90) in the ss-GKRS group and 54.0 Gy_3_ (range 46.7–66.7) in the hf-GKRS group. The hf-GKRS group had a lower calculated median BED_3_ despite having a higher median tumor volume.

### 3.2. Outcomes

**Figure 2:**
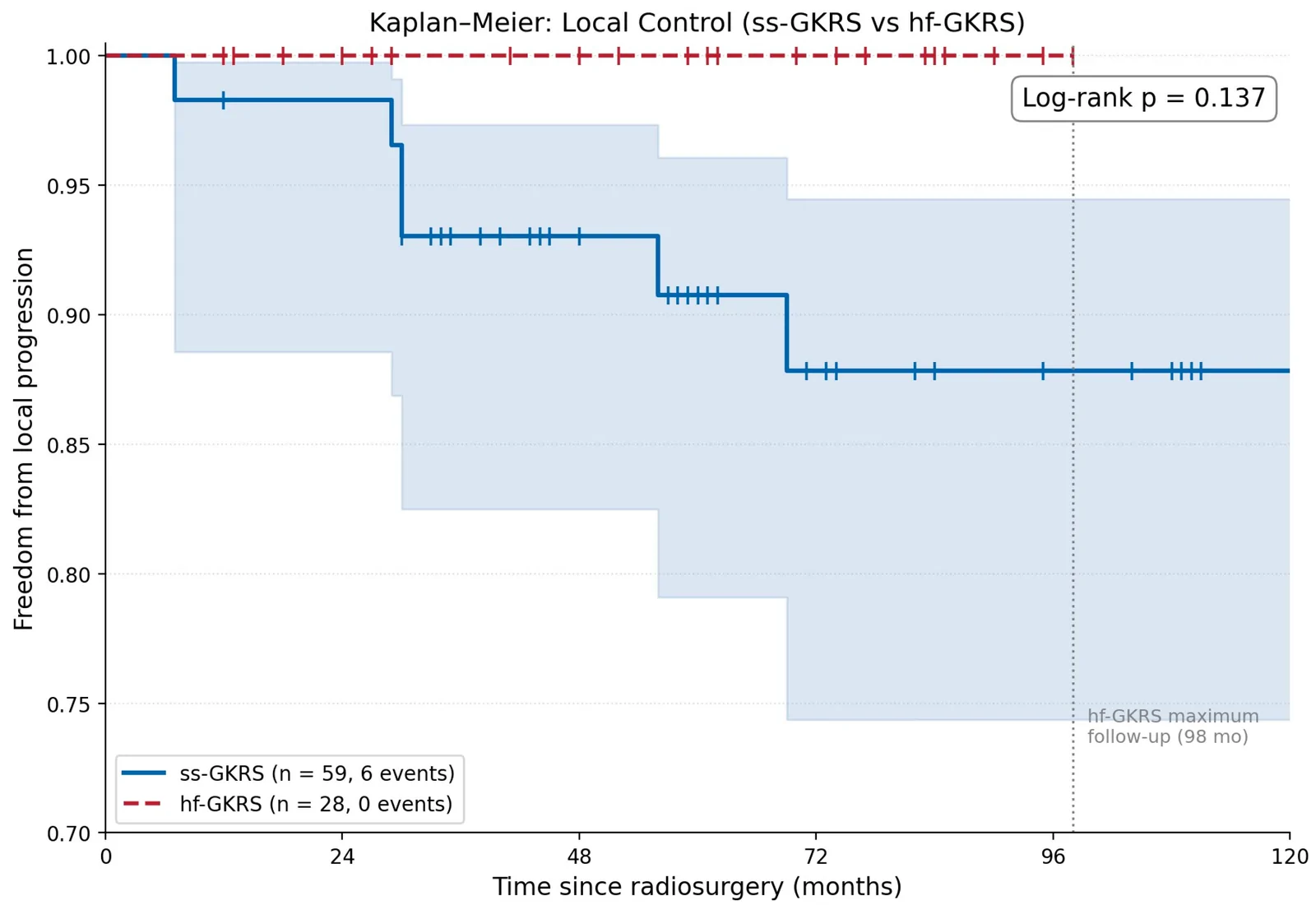
Kaplan–Meier estimates of freedom from local progression according to treatment modality. Tick marks indicate censored observations; the shaded region is the 95% confidence interval for the ss-GKRS group. Six local progression events occurred, all after ss-GKRS; because no events occurred after hf-GKRS, a confidence band could not be estimated. Deaths unrelated to PSM were censored at the date of death. The y-axis is truncated at 0.70 and the x-axis at 120 months; maximum follow-up was 186 months after ss-GKRS and 98 months after hf-GKRS (vertical dotted line). Log-rank *p* = 0.137.

Six LFs occurred across the cohort, all in the ss-GKRS group. Freedom from local progression did not differ significantly between treatment modalities (log-rank *p* = 0.137; Figure 2). One-, 3-, and 5-year estimates of freedom from local progression in the ss-GKRS group were 98%, 93%, and 91%, respectively. Among the six patients with LF, the median time to progression was 30 months (range 7–69 months), and three patients underwent surgery for progression.

Overall crude LC was 93.1% (81/87) across the cohort, 89.8% (53/59; exact 95% CI 79.2–96.2%) after ss-GKRS, and 100% (28/28; exact 95% CI 87.7–100%) after hf-GKRS. The exact 95% CI for LC after hf-GKRS was 87.7–100%, and maximum follow-up was shorter in the hf-GKRS group than in the ss-GKRS group (98 vs. 186 months).

Post-treatment radiological edema was observed in 30 patients (34.5%) across the cohort, and 12 patients (13.8%) developed new clinical symptoms. Among the 12 patients with new clinical symptoms, headache was most common (7/12, 58.3%), followed by seizures (4/12, 33.3%) and motor or sensory deficits (1/12, 8.3%). Radiological edema occurred in 23 of 59 ss-GKRS patients (39.0%; exact 95% CI 26.5–52.6%) and 7 of 28 hf-GKRS patients (25.0%; exact 95% CI 10.7–44.9%) (Fisher exact *p* = 0.235). Among patients without pre-treatment edema, new post-treatment edema occurred in 18 of 50 ss-GKRS patients (36.0%) and 2 of 11 hf-GKRS patients (18.2%). Among patients with pre-treatment edema, worsening of pre-existing edema occurred in 5 of 9 ss-GKRS patients (55.6%) and 5 of 17 hf-GKRS patients (29.4%).

**Figure 3:**
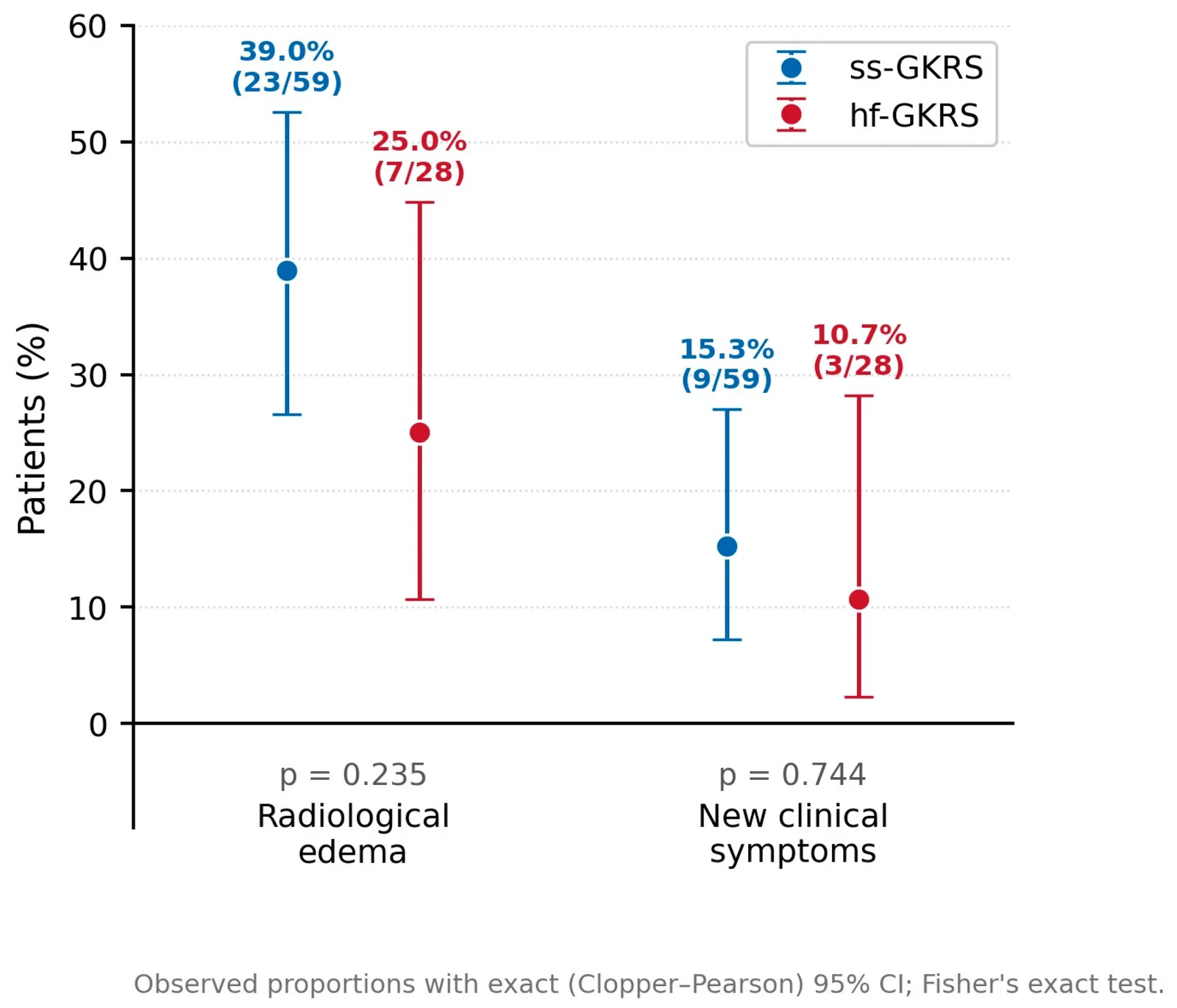
Post-treatment radiological and clinical events by treatment modality, shown as observed proportions with exact (Clopper–Pearson) 95% confidence intervals. Post-treatment radiological edema: ss-GKRS 39.0% (23/59, 95% CI 26.5–52.6) versus hf-GKRS 25.0% (7/28, 95% CI 10.7–44.9), Fisher exact *p* = 0.235. New clinical symptoms: ss-GKRS 15.3% (9/59, 95% CI 7.2–27.0) versus hf-GKRS 10.7% (3/28, 95% CI 2.3–28.2), *p* = 0.744 (Fisher’s exact test). Because median follow-up differed between groups (71.5 versus 60 months), these are crude proportions rather than time-dependent incidence rates.

**Figure 4:**
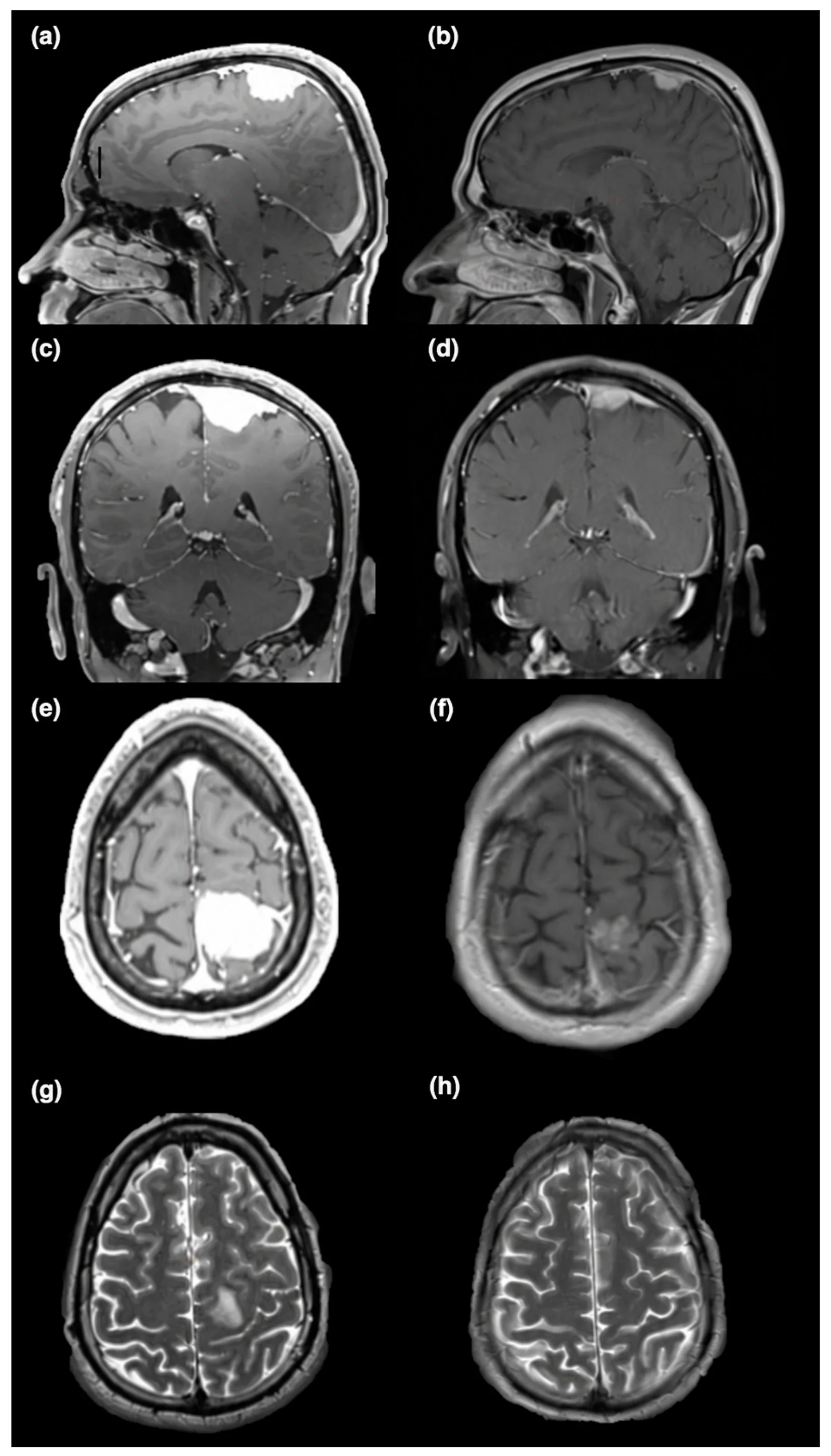
Representative long-term radiological outcome after hf-GKRS. A 31-year-old man with an 11.2 cm^3^ PSM treated with 18 Gy in 3 fractions prescribed to the 45% isodose line. (**a**,**c**,**e**) Pre-treatment sagittal, coronal, and axial contrast-enhanced T1-weighted MRI. (**b**,**d**,**f**) Corresponding images 69 months after hf-GKRS, showing marked tumor regression. (**g**) Pre-treatment axial T2-weighted MRI showing peritumoral edema. (**h**) Follow-up axial T2-weighted MRI showing resolution of the edema. No new clinical symptoms occurred during follow-up. Facial soft tissue has been masked on the sagittal images to prevent patient identification.

New clinical symptoms occurred in 9 of 59 ss-GKRS patients (15.3%; exact 95% CI 7.2–27.0%) and 3 of 28 hf-GKRS patients (10.7%; exact 95% CI 2.3–28.2%) (Fisher exact *p* = 0.744); all 12 patients with new clinical symptoms also had post-treatment radiological edema (Figure 3). Neither difference was statistically significant, and the confidence intervals overlapped substantially. Median time to symptom development was 5 months (range 2–30) in the ss-GKRS group and 2 months (range 1–7) in the hf-GKRS group. Two of the 12 patients with new clinical symptoms (16.7%), both in the ss-GKRS group, required surgical intervention, whereas the remaining 10 patients were managed medically with corticosteroids and/or antiseizure medication. A representative long-term radiological course after hf-GKRS is shown in Figure 4. Because median follow-up differed between groups, these are crude proportions rather than time-dependent incidence rates.

### 3.3. Exploratory Volume-Stratified Analysis

Tumor volume showed exploratory discrimination for LF, with an area under the curve of 0.766 (95% CI 0.538–0.994, *p* = 0.031). The cut point maximizing the Youden index was 11.75 cm^3^ (sensitivity 1.000, specificity 0.617); all six LFs occurred above this value. The confidence interval around the area under the curve was wide because only six LFs were available, and the 11.75 cm^3^ cut point was internally derived without independent validation; it should therefore be regarded as hypothesis-generating rather than as a validated clinical threshold.

In this exploratory stratification, crude LC was 100% in both groups for tumors ≤11.75 cm^3^ (ss-GKRS 38/38; hf-GKRS 12/12). Among tumors >11.75 cm^3^, crude LC was 71.4% (15/21; exact 95% CI 47.8–88.7%) after ss-GKRS and 100% (16/16; exact 95% CI 79.4–100%) after hf-GKRS (Fisher exact *p* = 0.027). These confidence intervals overlap, and because no failures occurred in the hf-GKRS subgroup, effect-size estimates were unstable and are not reported. Across the entire cohort, crude LC did not differ significantly between modalities (89.8% versus 100%, Fisher exact *p* = 0.171). These subgroups were small (hf-GKRS *n* = 12 at or below and *n* = 16 above the cut point), and a significant result within one volume stratum does not establish a treatment × volume interaction.

BED_3_ could not be evaluated independently of treatment modality. All six LFs occurred in the ss-GKRS group, which received a higher median BED_3_ (60.0 versus 54.0 Gy_3_) despite smaller tumors. Because BED_3_ distributions were strongly dependent on treatment modality and all LFs occurred in one treatment group, the independent association between BED_3_ and LC could not be reliably estimated; BED_3_ was therefore not entered into a multivariable model. BED_3_ was not significantly associated with post-treatment edema in univariable analysis (*p* = 0.305).

## 4. Discussion

The surgical management of PSMs remains challenging due to their relationship with the SSS and adjacent cortical veins. Complete resection often becomes infeasible without significant risk of neurological morbidity [5,23]. As a result, STR followed by adjuvant radiotherapy or radiosurgery has become an important management strategy when complete resection cannot be achieved safely [14,24,25]. Even with aggressive microsurgical approaches, recurrence rates as high as 13% have been reported, underscoring the need for durable adjuvant or alternative treatment options [3,26].

Radiosurgery has emerged as an important modality, either as primary treatment or after STR [27,28]. Pollock et al. [29] provided broader evidence supporting durable tumor control after SRS for meningiomas. More directly comparable to our cohort, Azar et al. [30] reported an LC rate of 91.8% after GKRS specifically for PSMs over a mean follow-up of 30 months. Our cohort showed an overall crude LC rate of 93.1% in large (≥8 cm^3^) PSMs.

PSMs are particularly prone to the development of peritumoral edema following radiosurgery [15,31]. In addition to vasogenic and cytotoxic mechanisms, transient post-SRS parenchymal swelling and venous compression may exacerbate this complication [32]. Prior reports, including those by Sheehan et al. [33] and Cai et al. [34], have identified tumor volume (>10 cc), venous sinus invasion, and the tumor–brain contact surface as significant predictors of post-radiosurgical edema. Given these well-established risk factors, treatment regimen is an important consideration when balancing tumor control against the risk of radiation-related toxicity.

Hypofractionated SRS has been developed to address these challenges, allowing multiple smaller fractions of radiation to be delivered over consecutive sessions [35]. The radiobiological rationale for hypofractionation is that delivery of smaller doses per fraction may allow for greater normal-tissue repair between sessions and thereby improve treatment tolerance [36]. Clinically, hypofractionated approaches have therefore been used for larger or critically located meningiomas in which single-session treatment may raise concerns regarding radiation-related toxicity [37].

In our cohort, new clinical symptoms occurred in 15.3% after ss-GKRS and 10.7% after hf-GKRS (*p* = 0.744). This non-significant difference should not be interpreted as evidence of equivalent safety, particularly given the small sample size and non-random treatment allocation. These findings are consistent with prior comparative analyses. Smith et al. [37], in a cohort of meningiomas >2 cm, reported higher five-year freedom from LF with hypofractionated SRS (91% versus 63.6%) without a significant increase in toxicity. Notably, this occurred despite a lower biologically effective dose in the hypofractionated group (20 Gy in 4 fractions, BED_3_ 53.3 Gy_3_) than in the single-session group (12 Gy, BED_3_ 60.0 Gy_3_), the same direction observed in our cohort (54.0 versus 60.0 Gy_3_). The similar direction of BED_3_ differences in the two series is noteworthy; however, neither study can determine whether the observed outcomes are attributable to fractionation itself, radiobiological dose, tumor characteristics, or treatment selection (Table 2). The present study adds a location-specific perspective by focusing exclusively on relatively large PSMs, in which venous sinus involvement and peritumoral edema represent considerations for radiosurgical treatment.

Post-treatment radiological edema remains an important consideration after stereotactic treatment of intracranial meningiomas, particularly in larger lesions and in patients with pre-existing risk factors [33,38]. Although baseline edema was substantially more frequent in the hf-GKRS group (60.7% vs. 15.3%), post-treatment radiological edema was not higher in this group (25.0% vs. 39.0%). This observation should remain descriptive because of the marked baseline imbalance and non-random treatment allocation. When stratified descriptively by baseline edema status, new edema developed in 36.0% of ss-GKRS patients and 18.2% of hf-GKRS patients without pre-treatment edema, whereas worsening of pre-existing edema occurred in 55.6% and 29.4%, respectively. Given the small subgroup sizes and non-random treatment selection, these findings should be regarded as descriptive and do not establish a protective effect of fractionation.

Radiological edema was not invariably accompanied by clinical symptoms. Of the 30 patients with post-treatment radiological edema, 12 (40.0%) developed new clinical symptoms; conversely, all 12 patients with new clinical symptoms had concurrent radiological edema. Two patients required surgical intervention, whereas the remaining 10 were managed medically. These findings underscore the importance of distinguishing radiological edema from clinical events, although a causal relationship between edema and the associated symptoms cannot be established from this retrospective dataset.

BED is a useful radiobiological parameter for comparing fractionation schedules and estimating the balance between tumor control and normal tissue toxicity [21,39]. In our cohort, as all progressions were observed in the ss-GKRS group, the relationship between BED and LC could not be meaningfully interpreted independently of treatment type. No significant association was observed between BED_3_ and post-treatment radiological edema. Because BED_3_ was strongly dependent on the selected fractionation regimen and all LFs occurred in the ss-GKRS group, its independent relationship with LC cannot be determined from this cohort.

Taken together, these findings suggest that hf-GKRS is a clinically feasible treatment strategy for selected patients with large PSMs, although superiority over single-session treatment cannot be established from the present observational data. ss-GKRS remains an established treatment option for appropriately selected meningiomas, while hf-GKRS may represent an alternative approach for larger or higher-risk tumors in which fractionation is considered clinically appropriate.

**Table 2:** Literature review: single-session and hypofractionated stereotactic radiosurgery for large meningiomas.

Author, Year (Ref.)	Design	Patients	Modality	Dose (Median)	Tumor Volume (Median)	Local Control	Follow-Up	Adverse Effects/Toxicity	Key Predictors
Single-Session Radiosurgery									
El-Shehaby et al. (2021) [12]	Retrospective cohort	273 (≥10 cc; 68% upfront SRS; 84% skull base)	GK-SS	Median 12 Gy (range 9–15 Gy)	Median 15.5 cc (range 10–57.3 cc)	5 y: 96%; 10 y: 81%; overall 90%	Median 6.1 y (range 2–18)	15% AREs; 6% symptomatic, all resolved with steroids	Female sex and longer follow-up associated with control; PFS <80% long-term for tumors ≥20 cc
Bledsoe et al. (2010) [17]	Retrospective cohort	116 (WHO grade I, >10 cm^3^; 64% prior surgery)	GK-SS	Median 15.1 Gy (range 12–18 Gy)	Mean 17.5 cc (range 10.1–48.6 cc)	3 y: 99%; 7 y: 92%	Mean 70.1 mo (range 12–199)	23% symptomatic complications; supratentorial 44% vs. skull base 18%	Supratentorial location: significantly higher complication rate vs. skull base
Ganz et al. (2009) [40]	Retrospective cohort	97 (meningiomas ≥10 cm^3^; 83% skull base)	GK-SS	12 Gy (reduced only to protect adjacent structures)	Mean 15.9 cc (range 10–43.2 cc)	100% at 2 y; 31% shrinkage	Mean 54 mo (range 25–86)	3%; all transient, resolved	Volume not related to complications when marginal dose kept at 12 Gy
Starke et al. (2015) [41]	Retrospective cohort	75 (skull base, >8 cm^3^; 60% prior surgery)	GK-SS	Mean 13.5 Gy (range 4.8–30 Gy)	Mean 14.1 cc (range 8.1–54.8 cc)	3 y: 90.3%; 5 y: 88.6%; 10 y: 77.2%	Mean 6.5 y (range 0.5–21)	15% new/worsened edema or CN neuropathy; neurological decline 17%	CN III–VI deficit, prior RT, and volume >14 cm^3^ associated with progression
Goyal-Honavar et al. (2024) [42]	Retrospective cohort	76 (>10 cc; 51.3% prior surgery)	GK-SS	Mean 12.5 Gy (range 6–15 Gy)	Mean 12.55 cc (range 10.3–25 cc)	5 y PFS: 72%; 20% radiological progression	Median 48 mo	High rate of subsequent surgery after progression; overall progression 20%	Volume >14 cc, limb motor deficits, and Simpson grade 3/4 excision associated with worse PFS
Taori et al. (2025) [43]	Retrospective (upfront SRS only)	112 (WHO grade I, no prior surgery)	GK-SS	12 Gy (range 10–15 Gy); max 24 Gy (range 20–30)	Median 13 cc (range 10–24.7 cc); V12: 13.9 cc	3 y: 98%; 5 y: 97%; 10 y: 88%; 14% progressed	Median 106 mo (range 24–307)	ARE 6%; peritumoral edema; managed with pentoxifylline (86%) ± bevacizumab (14%)	Volume >17 cc predicts worse LTC; supratentorial location increases ARE risk
Hypofractionated Radiosurgery									
Pinzi et al. (2023) [13]	Prospective phase 2 trial	166 (grade I; ≥3 cm and/or critically located; 49% prior surgery)	CK-HF (5 fx)	25 Gy in 5 daily fractions (89%)	Mean 14 cc (range 0.76–126 cc)	5 y: 97%; 7 y: 95%	Median 53 mo (range 4–101)	12.7% overall toxicity; 5% visual impairment; 4% trigeminal numbness	No significant toxicity predictors; pre-SRS symptoms, histology, site associated with LC (univariate)
Park et al. (2018) [44]	Retrospective (upfront only)	23 (skull base, ≥10 cm^3^, no prior treatment)	GK-HF (3–4 fx)	Median 18 Gy (range 15–20 Gy); 6 Gy/fx	Mean 21.6 cc (range 10.09–71.42 cc)	100% at mean 38 mo	Mean 38 mo (range 17–78)	17% transient CN neuropathy (resolved); 9% edema; 0% permanent deficit	No significant predictors identified (small cohort)
Oh et al. (2020) [45]	Retrospective cohort	31 (skull base, >10 cm^3^; 87% upfront)	CK-HF (median 5 fx, range 3–5)	Median 27.8 Gy (range 22.6–27.8 Gy)	Mean 18.9 cc (range 11.6–58.2 cc)	3 y: 94%; 5 y: 90%	Median 57 mo (range 9–98)	9.6% ARE; no permanent radiation-related deficit	WHO grade II histology only predictor of treatment failure
Song & Go (2024) [46]	Retrospective (preliminary)	18 (≥10 cm^3^; inoperable)	CK-HF (median 3 fx, range 3–5)	Median 24 Gy (range 21–30 Gy)	Median 14.8 cc (range 11.0–28.6 cc)	5 y: 93.8%; overall 94.4%	Median 30.5 mo (range 2–107)	66.7% symptomatic edema; 16.7% rescue craniotomy; 1 unrelated death	Small cohort; pseudoprogression observed; CKRS feasible for inoperable patients
Mixed Series (Single-Session & Hypofractionated)									
Smith et al. (2025) [37]	Retrospective cohort	172 (121 ss-SRS, 51 hSRS)	GK-SS & HF	SRS 12 Gy (IQR 12–13); hSRS 20 Gy/4 fx	SRS 6.1 cm^3^ (4.3–10.2); hSRS 15.9 cm^3^ (10.0–20.3)	FFLF-SRS: 87/79/63.6%; hSRS: 96/91/91% (1/3/5 y)	Median truncated at 80 mo	Any CTCAE-SRS 26.4%, hSRS 29.4%; Grade ≥2-SRS 14.9%, hSRS 23.5%	Toxicity not different after adjusting for volume; volume significantly predicts toxicity
Han et al. (2017) [47]	Retrospective (comparative)	70 (42 ss-GKRS, 28 hf-GKRS)	GK-SS vs. HF (2–4 fx)	SS 12 Gy (8–14); HF 7.5 Gy × 2/6 Gy × 3/4.5 Gy × 4	SS 15.2 cc; HF 21 cc	5 y: 88.1% (SS) vs. 92.9% (HF); *p* = 0.389	SS 57.8 mo; HF 50 mo	SS 33.3%; HF 7.1%; *p* = 0.017	Fractionation: significantly lower complication rate (HR 5.7:1, *p* = 0.017)
Pahwa et al. (2025) [48]	Systematic review & meta-analysis (13 studies)	1064 tumors (791 SSRS 74%, 273 HSRS 26%; >8 cc)	GK, CK-SS & HF	SSRS median 13.18 Gy; HSRS variable (3–5 fx)	Overall 16 cc; SSRS 14.6 cc; HSRS 17.6 cc	Pooled: 90%; SSRS: 90%; HSRS: 95%	Overall 58.7 mo; SSRS 67 mo; HSRS 49 mo	Pooled PTE 7.9% (95% CI 5.5–11.2%); SSRS 8.0%; HSRS 7.2%	HSRS achieves outcomes similar to SSRS; skull base tumors more common in HSRS
Present study	Retrospective cohort	87 (59 ss-GKRS, 28 hf-GKRS; 41.4% prior surgery)	GK-SS & HF (3/5/10 fx)	ss-GKRS 12 Gy (10–15); hf-GKRS 20 Gy total (18–30)	Median 11.2 cm^3^ (8–37.8); ss 10.6 cm^3^; hf 14.1 cm^3^	Crude LC: overall 93.1%; ss 89.8%; hf 100%; >11.75 cm^3^-ss 71.4% vs. hf 100%	Median 62 mo (range 12–186); ss 71.5 mo, hf 60 mo	Post-treatment radiological edema 34.5% (ss 39.0%, hf 25.0%); clinical symptoms 13.8% (ss 15.3%, hf 10.7%)	Exploratory volume analysis suggested lower LC with increasing tumor volume; the 11.75 cm^3^ cut point was internally derived and not validated

ARE, adverse radiation effect; CK, CyberKnife; CN, cranial nerve; CTCAE, Common Terminology Criteria for Adverse Events; FFLF, freedom from local failure; fx, fractions; GK, Gamma Knife; HF/HSRS, hypofractionated stereotactic radiosurgery; LC, local control; LTC, long-term control; PFS, progression-free survival; PTE, peritumoral edema; Ref., reference number in the manuscript reference list; RT, radiotherapy; SS/SSRS, single-session stereotactic radiosurgery; SRS, stereotactic radiosurgery; WHO, World Health Organization. Within each category, studies are ordered by ascending reference number.

### Limitations

Several limitations should be emphasized. First, only 6 local-failure events were observed across the cohort; this severely limits the precision and stability of the ROC-derived volume threshold and any subgroup comparisons, all of which should be regarded as exploratory rather than confirmatory. Second, the study spans a 19-year period (2006–2025), during which Gamma Knife hardware, treatment-planning software, imaging quality, and clinical practice may have changed. hf-GKRS was introduced in 2018, whereas ss-GKRS was used throughout the study period; therefore, treatment modality may be partially confounded by treatment era, although the substantial temporal overlap between the two modalities limits complete separation by era. Third, treatment allocation was non-random, and selection for hf-GKRS was explicitly influenced by tumor volume, proximity to eloquent cortex, degree of sinus involvement, pre-treatment edema, and estimated radiation-related risk. This resulted in substantial confounding by indication, as reflected by baseline differences in prior surgery, pre-treatment edema, age, and tumor volume. Detailed extent-of-resection data and classification of radiosurgical targets as postoperative residual versus recurrent disease were not available for the 36 previously operated patients and therefore could not be compared between treatment groups. In addition, because the post-treatment radiological edema endpoint comprised both new edema and worsening of pre-existing edema, the marked imbalance in baseline edema further limits direct comparison of crude edema rates between treatment groups. Given the small number of LFs, this confounding could not be reliably addressed with multivariable adjustment, and comparative differences between treatment groups should therefore not be interpreted as causal effects of fractionation. Fourth, the hf-GKRS group included heterogeneous fractionation schedules (3 × 6 Gy, 5 × 4 Gy, 5 × 4.5 Gy, 5 × 5 Gy, and 10 × 3 Gy), with differing total doses and BED_3_ values, but these regimens were analyzed as a single group. The small number of patients treated with each schedule precluded meaningful regimen-specific outcome comparisons. Finally, the 11.75 cm^3^ volume threshold was derived and tested within the same dataset without independent or bootstrap validation and should not be regarded as a validated clinical decision threshold.

## 5. Conclusions

In this retrospective cohort, no LFs were observed after hf-GKRS during a shorter follow-up period, whereas 6 LFs occurred after ss-GKRS; the overall time-to-failure comparison did not reach statistical significance. These findings suggest that hf-GKRS is a feasible treatment option for selected patients with larger PSMs. However, neither superiority of hf-GKRS over ss-GKRS nor a definitive tumor-volume threshold can be established from these retrospective data, and prospective multicenter studies are needed for validation.

## Figures and Tables

**Table 1:** Patient, tumor, and treatment characteristics.

Characteristic	All Patients (*n* = 87)	ss-GKRS (*n* = 59)	hf-GKRS (*n* = 28)
Demographics			
Age (years), median [range]	57 (20–82)	59 (23–82)	49 (20–77)
Sex, female	53 (60.9%)	36 (61.0%)	17 (60.7%)
Sex, male	34 (39.1%)	23 (39.0%)	11 (39.3%)
Clinical Presentation			
Headache	40 (46.0%)	26 (44.1%)	14 (50.0%)
Seizure	29 (33.3%)	20 (33.9%)	9 (32.1%)
Motor/sensory deficit	9 (10.3%)	6 (10.2%)	3 (10.7%)
Gait disturbance	3 (3.4%)	3 (5.1%)	—
Incidental	4 (4.6%)	3 (5.1%)	1 (3.6%)
Aphasia	1 (1.1%)	—	1 (3.6%)
Visual impairment	1 (1.1%)	1 (1.7%)	—
Tumor Characteristics			
Prior surgical resection	36 (41.4%)	18 (30.5%)	18 (64.3%)
Pre-treatment edema	26 (29.9%)	9 (15.3%)	17 (60.7%)
Tumor localization			
Anterior third	18 (20.7%)	12 (20.3%)	6 (21.4%)
Middle third	64 (73.6%)	43 (72.9%)	21 (75.0%)
Posterior third	5 (5.7%)	4 (6.8%)	1 (3.6%)
Sindou classification			
Type I	22 (25.3%)	15 (25.4%)	7 (25.0%)
Type II	10 (11.5%)	7 (11.9%)	3 (10.7%)
Type III	3 (3.4%)	2 (3.4%)	1 (3.6%)
Type IV	6 (6.9%)	3 (5.1%)	3 (10.7%)
Type V	14 (16.1%)	10 (16.9%)	4 (14.3%)
Type VI	32 (36.8%)	22 (37.3%)	10 (35.7%)
GKRS Parameters			
Tumor/treatment volume (cm^3^), median [range]	11.2 (8–37.8)	10.6 (8–32)	14.1 (8–37.8)
Total prescription dose (Gy), median [range]	—	12 (10–15)	20 (18–30)
Dose per fraction (Gy), median [range]	—	12 (10–15)	4 (3–6)
BED_3_ (Gy_3_), median [range]	60.0 (43.3–90)	60.0 (43.3–90)	54.0 (46.7–66.7)
Dose regimen			
Single fraction (10–15 Gy)	59 (67.8%)	59 (100%)	—
3 × 6 Gy	7 (8.0%)	—	7 (25.0%)
5 × 4 Gy	13 (14.9%)	—	13 (46.4%)
5 × 4.5 Gy	2 (2.3%)	—	2 (7.1%)
5 × 5 Gy	4 (4.6%)	—	4 (14.3%)
10 × 3 Gy	2 (2.3%)	—	2 (7.1%)
Outcomes			
Follow-up (months), median [range]	62 (12–186)	71.5 (12–186)	60 (12–98)
Death from unrelated cause	8 (9.2%)	6 (10.2%)	2 (7.1%)
Crude local control (LC)	93.1% (81/87)	89.8% (53/59)	100% (28/28)
LC, tumors ≤11.75 cm^3^	100% (50/50)	100% (38/38)	100% (12/12)
LC, tumors >11.75 cm^3^	83.8% (31/37)	71.4% (15/21)	100% (16/16)
Post-treatment radiological edema	34.5% (30/87)	39.0% (23/59)	25.0% (7/28)
New clinical symptoms	13.8% (12/87)	15.3% (9/59)	10.7% (3/28)
Headache	7	5	2
Seizure	4	3	1
Motor/sensory deficit	1	1	—

BED_3_, biologically effective dose (α/β = 3 Gy); GKRS, Gamma Knife radiosurgery; hf-GKRS, hypofractionated Gamma Knife radiosurgery; LC, local control; ss-GKRS, single-session Gamma Knife radiosurgery. Percentages are column percentages calculated from the corresponding group denominator (all patients *n* = 87, ss-GKRS *n* = 59, hf-GKRS *n* = 28), except where an explicit numerator/denominator is shown. The 11.75 cm^3^ cut point is exploratory and internally derived (see Section 2.5). Em dash indicates not applicable or no patients in that category.

## Data Availability

The original contributions presented in this study are included in the article. Further inquiries can be directed to the corresponding author.

## Abbreviations

The following abbreviations are used in this manuscript:

ARE	adverse radiation effect
BED	biologically effective dose
CI	confidence interval
CK	CyberKnife
CT	computed tomography
GKRS	Gamma Knife radiosurgery
hf-GKRS	hypofractionated Gamma Knife radiosurgery
LC	local control
LF	local failure
LINAC	linear accelerator
MRI	magnetic resonance imaging
PSM	parasagittal meningioma
ROC	receiver operating characteristic
SRS	stereotactic radiosurgery
ss-GKRS	single-session Gamma Knife radiosurgery
SSS	superior sagittal sinus
STR	subtotal resection
WHO	World Health Organization
